# Toward an expansion of an enactive ethics with the help of care ethics

**DOI:** 10.3389/fpsyg.2014.01354

**Published:** 2014-11-27

**Authors:** Petr Urban

**Affiliations:** Department of Contemporary Continental Philosophy, Institute of Philosophy, The Academy of Sciences of the Czech RepublicPrague, Czech Republic

**Keywords:** enactivism, enactive ethics, feminist relational theory, the ethics of care, socially extended mind

## Introduction

An important and urgent way of widening the scope of embodied and situated approaches to intersubjectivity consists in exploring their implications for ethics^1^. Cash (2010, 2013) has recently argued for a rethinking of seminal ethical concepts against the background of the idea of *socially distributed* cognition. Colombetti and Torrance (2009) have proposed an ethics based on an *enactive* cognitive science of social life^2^. In this short paper, I want to focus mainly on the latter proposal and argue that recent developments in the enactive approach to social phenomena call for further expansion of an enactive ethics beyond its initial focus on face-to-face dyadic interactions. In this respect I aim to draw attention to the so far underappreciated kinship between an enactive ethics and the ethics of care. I consider the alliance of these two as remarkably well suited for abandoning the pitfalls of a widespread view of human autonomy in terms of the self-determination of individual rational agents, a view that has been systematically questioned from the perspective of care ethics over the last 35 years, but which still exerts a strong influence on our thinking about the good life and morality^3^.

## Enactive ethics and socially extended mind

Colombetti and Torrance (2009) made the first attempt—and the only one that has been made thus far—to show that the enactivist shift of attention from the individual to the interactional and relational domain (De Jaegher and Di Paolo, 2007) has profound repercussions for *ethics*^4^. What each of us does in relation to another is to be structured and characterized, according to the enactive view, primarily in inter-individual and interpersonal terms. It can be said from this perspective that the ethical character of a given situation arises, at least in part, from the meanings that emerge out of the inter-relations between the participants. These ideas suggest several important shifts in moral theory. An enactive ethics invites us to explore “the deep ethical ramifications of the participatory, collective dynamics of human inter-relations *per se*, as opposed to the ethical significance of individual actions and their simple aggregations” (Colombetti and Torrance, 2009, p. 517). It recommends a de-emphasis of the notions of individual autonomy and responsibility. The main lesson to be taken from the proposal of an enactive ethics is, thus, that the inter-relational, interactional, and inter-affective dimensions have to gain a central place in ethics, lest ethical theory overlook the very subject of its inquiry.

Colombetti and Torrance, however, have based their proposal of an enactive ethics on De Jaegher and Di Paolo's account (2007) and limited the scope of their inter-relational interpretation of moral phenomena exclusively to dyadic and face-to-face interactions. The most recent developments within the enactive approach to social life, however, transcend the narrow realm of dyadic interactions and open enactive research to a wider sphere of interactions with socio-cultural *institutions* (Steiner and Stewart, 2009; Froese and Di Paolo, 2011; Torrance and Froese, 2011). Human “sense-makers” construct shared meanings in their on-going interactions within the context of a vast array of social givens (Torrance and Froese, 2011, p. 45). The agent's entrance into an interactional and properly social domain requires abiding by a heritage of pre-established social and cultural norms, while at the same time expanding possibilities of the agent's sense-making and agency (Torrance and Froese, 2011). If we want to take into account the wider social and institutional dimension of social life as approached from the enactive perspective, the following urgent question seems inevitable: What would an appropriate expansion of an enactive ethics look like?

The wider normative dimension of social life plays a central role in a recent parallel attempt to reinterpret fundamental ethical concepts against the background of a different^5^ embodied and situated approach to cognition, which can be found in Cash (2010, 2013). Cash introduces the “third-wave arguments” for socially and culturally distributed cognition and distinguishes them from the individual-centered focus of the previous arguments for the extended mind hypothesis, which are based on Clark and Chalmers' pioneering work (Clark and Chalmers, 1998). Cash explores the implications of the idea of socially distributed cognition for seminal moral concepts, such as autonomy, agency, and responsibility. The main novelty of his answers, on my view, consists in the recommendation that the advocates of socially distributed cognition should avoid reinventing the wheel and avail themselves of extant arguments elaborated in principally feminist relational theory and criticism of the individualistic conceptions of self, agency, and moral autonomy. Cash refers in particular to the concepts of *relational autonomy* and *relational self* as introduced in the 1990s by feminist theorists and ethicists, such as Meyers (1989, 1997, 1998), Friedman (2000), Mackenzie and Stoljar (2000), and others, who argue, in general, that one's self and one's autonomy are decentralized and are relationally and socially constituted.

## Enaction and care ethics

I argue that the feminist relational theory, to which Cash's arguments appeal, and in particular the closely related *ethics of care* [as developed by Gilligan (1982), Noddings (1982); Ruddick (1989), Held (1993, 2006); Tronto (1993), Kittay (1999), and many others] can be considered as a rich source for further developing and expanding an enactive ethics. Both the enactive approach and the ethics of care attempt to rethink the concepts of autonomy, individuality and agency in a way that enables a novel reading of human relations in terms of the irreducibility of the inter-relational and interactional domain. On both approaches, agents are conceived as essentially embodied, situated, and embedded in multiple relational networks at different levels, from the biological to the social and the cultural level (e.g., Hamington, 2004). Concern and emotionality are central to both perspectives and are considered as part and parcel of any agents' making sense of the world and others (e.g., Held, 2006, pp. 21–22). However, the ethics of care undertook the shift to the interactive and interpersonal moral phenomena decades before a proposal of an enactive ethics had first been made. I argue that the conceptual and methodological toolkit of the ethics of care, its elaborated accounts of human interdependency, mutuality, engagement with social and political institutions, etc., should serve as a well-suited means of arriving at an appropriately expanded enactive view of social and moral phenomena. The experiential knowledge of the ethics of care, its sensitivity to the inequalities of power-relations and its developed views of complex structures and relations at various levels of human social life can provide useful tools for widening an enactive ethics to the broader domain of properly social life.

On the other hand, the enactive approach to social phenomena, based on the concept of participatory sense-making, provides a detailed description of the complex relations between persons, and between persons and institutions, which can help to account not only for the specific nature and dynamics of the social interdependence between persons (in terms of interactional autonomy), but also for the generation and subsistence of social institutions. Human social interactions are essentially situated in a normative context and are governed by various social institutions that make these interactions possible. However, these norms and institutions “don't just exist in a special normative realm independently of the actual lives of people: they are embedded in the ways people conduct those lives—their continued existence requires that they be continually (inter-) enacted, in either word or deed” (Torrance and Froese, 2011, p. 46). Real social interactions involve interpretation and sometimes even creative reinterpretation and modification of the very norms that are the framework within which they take place. The enactive look at “the origin of and fluid changes in normativity” (De Jaegher, 2013, p. 22) with the corresponding focus on the bi-directionality of influence between social interactions and social institutions, can help us explain how a criticism and transformation of social structures, institutions, and norms can materialize. And this is precisely what has been at stake in the ethics of care since soon after its conception (e.g., Held, 1993, 2006; Tronto, 1993, 2013; Sevenhuijsen, 1998; Engster, 2007; Barnes, 2012).

In this connection De Jaegher (2013) aims to show that we should consider the enactive approach as a better way of arriving at a full-blown picture of our interactions with social norms as compared to the proposal of socially extended and distributed cognition (as developed by e.g., Gallagher and Crisafi, 2009; Gallagher, 2013). On her view, the socially extended mind approach is limited to addressing rule-based, hierarchical institutions and interactions, and unable to grasp fluid and more participatory aspects of society. She holds this view, for she sees some aspects of the socially extended mind approach as being in line with functionalism of mainstream cognitive science, which deals with cognitive agents that are primordially lone individuals, instrumentally extending their “cognitive reach.” This is why the socially extended mind approach, according to her reading, tends to be one-sidedly focused on the functioning of ready-made, rigid normative systems, and therefore “would hardly tell us how institutions could be criticized or changed” (De Jaegher, 2013, p. 22).

This observation, if correct, indicates an important reason why the potential alliance between *enactivism* and care ethics may be seen as more promising and fruitful than the alliance between the theory of *socially distributed cognition* and feminist accounts of relational autonomy. However, we should proceed with caution and not overlook the fact that De Jaegher's criticism is aimed at the funcionalist and individualist core of the notion of a socially *extended* cognition (and only at Gallagher's and Crisafi's account to the extent that some elements of this view are still present in it). Most of her points would obviously not apply to the aforementioned “third-wave arguments” for socially and culturally distributed cognition (Cash, 2013). I deem it plausible to claim that the expansion of an enactive ethics with the help of care ethics, which I was arguing for in this paper, and Cash's proposal of an alliance between feminist relational theory and socially distributed cognition can and should be viewed as complementary rather than conflicting.

### Conflict of interest statement

The author declares that the research was conducted in the absence of any commercial or financial relationships that could be construed as a potential conflict of interest.

## Footnotes

^1^In this paper, the term *ethics* stands for moral theory, especially moral *philosophy*.

^2^In what follows, I will focus exclusively on the enactivist tradition whose philosophical foundations have been laid by Varela, Thompson, and Rosch in *The Embodied Mind* (Varela et al., 1991) and which has been further exemplified by Thompson (2005, 2007), Di Paolo (2005, 2009), De Jaegher and Di Paolo (2007), Di Paolo et al. (2010), and Froese and Di Paolo (2011).

^3^For a discussion of the general influence of individualistic views of autonomy on the current social, cultural and moral imaginary see e.g., Fineman (2004). Sass (2011) shows that the idea of individual autonomy—in terms of self-direction and volition—still plays the role of a widely recognized standard for assessing mental health. He characterizes it as an “extremely influential notion” in the field of psychopathology (Sass, 2011, p. 99). Ho (2008) puts forward a criticism of its predominant role in the field of bioethics, whereas Herring (2014) explores its current impact in the realm of law, in particular family law.

^4^It was not the first and only attempt to focus on the ethical implications of enactivism as such. Varela himself has done the pioneering work in his 1999 book on ethics (Varela, 1999). Recently, DeSouza (2013) has developed Varela's idea of the “ethical know-how” and brought it in connection in an interesting way with the current debates on the second nature. Nishigaki (2006) has elaborated the link between enactivism and ethics from a different perspective. He attempted to demonstrate an affinity between the enactive approach and Eastern ethical traditions. However, none of the above-mentioned authors have focused on the moral significance of the interactional and inter-relational domain as described in the enactive account of social life. Their focus has by and large been to put forward a novel view of the relationship between emotional and cognitive dimensions of moral sense-making.

^5^It has been repeatedly argued that the extended mind hypothesis (even the socially extended one) and enactivism are incompatible for a number of important reasons (e.g., Di Paolo, 2009; Thompson and Stapleton, 2009; Wheeler, 2010; De Jaegher, 2013). However, there are also a number of commonalities between the two approaches that allow us to qualify both of them as embodied and situated accounts of cognition and that justify the next step of our argumentation.
